# Determinants of institutional care willingness among Chinese older adults: an Andersen’s model-based analysis in Jiangning District, Nanjing

**DOI:** 10.3389/fpubh.2025.1692816

**Published:** 2025-10-13

**Authors:** Ling Zong, Zhengfang Chen

**Affiliations:** ^1^School of Health, Jiangsu Vocational Institute of Commerce, Nanjing, Jiangsu, China; ^2^School of Public Administration, Hohai University, Nanjing, Jiangsu, China

**Keywords:** older adults, institutional care, care willingness, influencing factors, Andersen’s model

## Abstract

**Introduction:**

By comprehensively examining the acceptance and preferences of older adults toward institutional care, and conducting an in-depth analysis of the various factors that influence these attitudes, we can gain a clearer understanding of their care needs. This insight will help deliver more appropriate and personalized care services and provide a scientific foundation for governments and social organizations to develop relevant policies and service programs.

**Methods:**

A sample of older adults from Jiangning District, Nanjing, was selected as the research subjects. The questionnaire was designed based on the Andersen’s Model, and a multi-stage mixed sampling method was adopted to investigate older adults’ willingness toward institutional care. Additionally, a binary logistic regression model was employed to analyze the factors influencing their willingness.

**Results:**

A total of 415 valid survey data were collected, among which 44% of the surveyed older adults expressed willingness to choose institutional care. Correlation analysis revealed that there are key influencing factors within predisposing factors, enabling factors, and need factors, all of which exert an impact on the willingness toward institutional care. Further logistic regression analysis indicated that factors such as stigma associated with institutional care, participation in endowment insurance, purchase of medical insurance, perceived attitudes of children toward care institutions, accessibility of resources in care institutions, self-rated physical health status, and presence of chronic diseases may be influencing factors of the older adults’ willingness toward institutional care.

**Conclusion:**

Compared with other regions in China, older adults in Jiangning currently have a relatively high willingness toward institutional care, which is influenced by multiple factors. Specifically, after incorporating predisposing factors, enabling factors, and need factors simultaneously into the model, older adults who held an open attitude toward institutional care, had endowment insurance and medical insurance, perceived their children’s positive attitudes toward care institutions, had easier access to institutional care resources, reported relatively good self-rated physical health, and had no chronic diseases demonstrated a stronger willingness toward institutional care.

## 1 Introduction

As noted in the United Nations’ World Population Prospects 2024, the global population aging is evolving at an accelerating pace, with the world population projected to reach a peak of approximately 1.03 billion around the mid-2080s. The report specifically emphasizes that the growth rate of the global population aged 65 and above will significantly outpace that of the population under 18, and this trend will be particularly prominent by the end of the 2070s (1). To address the rapid progression of population aging, SDG 3, SDG 10, and SDG 11 within the United Nations’ 2030 Agenda for Sustainable Development (SDGs) all focus on aging-related issues. Specifically, they emphasize access to essential healthcare services for age groups including the older adults, as well as the development of age-friendly public facilities for aged care. Article 12 of the World Population Plan of Action (1970s) explicitly states that the demographic structure of developing countries is shifting toward aging. It therefore calls on governments to pay attention not only to fluctuations in fertility rates but also to the growth of the older population, and to integrate social security for the older adults into national overall development plans (2). Thus, population aging constitutes a global issue. The growing older population not only imposes higher demands on the aged care care service system but also presents pressing social challenges (3). Against the backdrop of its substantial population base, China has become one of the countries worldwide experiencing the most rapid population aging and hosting the largest older population. Per the United Nations’ criteria for defining an aging society, a region is formally designated as entering an aging phase when the share of its population aged 60 years and above exceeds 10% of the total population, or when the proportion of those aged 65 years and above reaches 7%. A nation is deemed to have entered a “moderately aging” society if the percentage of people aged 60 years and above surpasses 20%, or if that of individuals aged 65 years and above exceeds 14%. An in-depth analysis of data from China’s Seventh National Population Census indicates that the population aged 60 years and above in China has reached 264 million, representing a notable 18.70% of the total population. Concurrently, the number of people aged 65 years and above stands at 191 million, with their share of the total population rising to 13.50% (4). This indicates that China is on the verge of entering a “moderately aging” society. It is projected that by 2050, the older population in China will approach the 500 million mark, signifying that the country’s population aging phenomenon will continue to intensify and exert far-reaching impacts on all aspects of society.

Population aging poses two core challenges to society. On one hand, older adults face issues regarding the sustainability of health security, social welfare, and care support systems. On the other hand, the younger generation is burdened with the economic and social pressures of supporting and caring for the older population. In China, influenced by traditional Confucian filial piety, family-based care—a traditional model with profound historical roots—remains the most prevalent and central choice for Chinese older adults. Its stable status and significance cannot be overlooked (5). Compared with other models, institutional care is typically adopted as an alternative option when older adults face specific predicaments or needs (6). However, under the influence of the one-child policy in the past, the number of children in most families has decreased, resulting in a widespread miniaturization of family structures (4). When these children enter marriage, they face the challenge of the “4-2-1” family structure, where two adults are required to care for four older individuals simultaneously. This family structure imposes a dual burden—both economic and energetic—on the younger generation, particularly when they assume the responsibility of family-based care.

Against the backdrop of the prevailing caregiving stress in one-child families, institutional care, as a socialized care model, is gradually highlighting its advantages. In urban areas, the economic independence of older adults is increasingly strengthened (7). Research data indicate that, provided their housing conditions and physical health permit, the majority of older adults tend to prefer aged care models where they live independently or only with their spouses (8, 9). Against this backdrop of change, some older adults choose to move out of their homes and relocate to various aged care service institutions that integrate medical care and aged care, such as profit-oriented or public welfare nursing homes and welfare centers, to receive more professional and systematic care and services. This not only meets their basic daily needs but also provides them with spiritual solace, effectively compensating for the limitations and inadequacies of other aged care models in terms of resource allocation and service provision. The rise of institutional care not only helps alleviate the pressure of family-based care but also promotes the further improvement and maturation of the aged care service system (10).

However, influenced by traditional culture and family values, the family-based aged care model has long dominated in countries such as China, Japan, South Korea, and Russia. Within the cultural contexts of these nations, providing family care for the older adults is regarded as a moral obligation, whereas opting for institutional care is often stigmatized as “unfilial” (11). Against the backdrop of such social perceptions, coupled with the objective limitations of aged care institutions in terms of service quality and living environments, these institutions hold relatively low appeal for the majority of older adults. This has resulted in both low willingness among older adults to move into such facilities and low actual occupancy rates. Currently, the most pressing societal issue is how to fully leverage the role of aged care institutions as a core pillar in the aged care service system to alleviate caregiving pressures, while exploring effective strategies to enhance older adults’ willingness to choose institutional care.

Currently, some studies have explored the impact of institutional care demand from dimensions such as social culture, national policies, and individual mental health (12, 13). The general acceptance of institutional care among China’s older population remains low, with significant disparities observed. At the individual level, variables such as age, educational attainment, marital status, self-care ability, and living arrangements exert significant impacts on care preferences. Notably, educational attainment exhibits a positive correlation with acceptance levels (14, 15). However, existing studies predominantly focus on the analysis of single factors, lack a systematic examination of the interaction effects among various influencing factors, and have not yet established a complete theoretical framework to explain the mechanism of the joint action of multiple factors.

Therefore, under the framework of the existing Andersen’s Behavioral Model of Health Services Use, this study elaborates on how multiple factors systematically influence the older adults’ willingness toward institutional care. This model has been applied in several Chinese studies, as follows: Hongjuan Liu explored the older adults’ preferences for long-term care models—including home care, home- and community-based care, and institutional care—and their influencing factors based on the Andersen Behavioral Model, and conducted a classified comparison of the factors affecting these three care models (16). Wang et al. (17) focused on the actual utilization of institutional care among disabled older individuals and examined its influencing factors, currently, the utilization of institutional care by disabled older adults remains extremely low. Zhou et al. (18) applied the Andersen’s Model to test the factors influencing urban residents’ preference for home-based care. Yang et al. (19) emphasized the impact of multiple factors on the willingness toward institutional care among disabled older individuals. Compared with existing studies, this study has three distinct strengths: first, it focuses more explicitly on the willingness toward institutional care itself, rather than extending the scope to other care models for older adults or actual care utilization behaviors; second, its research participants include both urban and rural older adult groups as well as ordinary older individuals with varying health statuses, addressing the limitation of narrow sample representativeness in previous research; third, it integrates localized innovations into the classic theoretical framework. Specifically, within the Chinese cultural context, the study incorporates the influence of cultural values, thereby providing a novel and more explanatory analytical perspective for understanding the dilemma of care decision-making among Chinese older adults.

As a key city in China’s eastern coastal region, Nanjing is also experiencing a gradual deepening of population aging. According to data from the reply by the Jiangning District Government of Nanjing to Proposal No. 0075 at the Second Session of the 13th Jiangning District Committee of the Chinese People’s Political Consultative Conference, the aging rate in Jiangning District stands at 18.28% (20). The aging of Jiangning District has led it to confront care challenges similar to those faced nationwide by older adults. Understanding the willingness of older residents in Jiangning District to choose institutional care, as well as the influencing factors behind such willingness, holds significant practical implications for meeting the older adults’ care needs and improving the quality of care services for older adults.

This study focuses on the older adult population in Jiangning District, Nanjing, aiming to comprehensively analyze their basic characteristics and acceptance willingness toward institutional care, and further explore the factors influencing such willingness. To enhance the theoretical depth and innovativeness of the research, this study will introduce and apply Andersen’s Behavioral Model of Health Services Use as the theoretical framework. Proposed by American medical sociologist Andersen, this model was initially applied to analyze individuals’ health care service utilization behaviors and has gradually evolved into a comprehensive and mature multi-dimensional framework (21). It primarily comprises four dimensions that constitute the research framework for individual health care accessibility, namely personal characteristics, health care behaviors, health care outcomes, and contextual characteristics. Among these, personal characteristics include predisposing factors, enabling factors, and need factors—all of which are the decisive factors for studying individuals’ health care service utilization and play the most critical role in the Andersen’s Model. Among numerous models of health care service utilization behaviors, the Andersen’s Model is widely recognized as the most comprehensive theoretical framework. It has also been extended to the field of care services for older adults and gained extensive application. In this study, based on questionnaire surveys on the three dimensions of the older adults’ personal characteristics and their willingness toward institutional care, and combined with an analysis of contextual characteristics such as local policies and culture, the influence of these factors on the older adults’ willingness toward institutional care was examined. This study aims to provide a more scientific and rational reference for aged care institutions in Nanjing, thereby promoting the optimization and improvement of care services for older adults.

## 2 Materials and methods

### 2.1 Study setting and study population

This study selected older adults from Jiangning District, Nanjing, as the survey respondents. To ensure the comprehensiveness and impartiality of the research, while considering the feasibility and efficiency of implementation, a multi-stage mixed sampling method was adopted. First, 4 sub-districts were randomly selected from 10 sub-districts using equal-probability sampling; Second, within each selected sub-district, 5 communities were further randomly sampled via equal-probability sampling, resulting in a total of 20 communities; Finally, in the selected communities, convenience sampling was used to select 22 households per community. A total of 440 questionnaires were collected initially, and after excluding invalid ones, 415 valid samples were obtained, with an effective response rate of 94.32%.

### 2.2 Data collection

In this study, a questionnaire survey method was employed to accurately capture the willingness of older adults in Jiangning District, Nanjing, to choose institutional care. This method, via a pre-designed questionnaire, aimed to collect respondents’ perspectives and circumstances regarding specific issues. Prior to conducting the survey, the study ensured that each participating older adults had explicitly expressed their willingness to take part. For those older adult respondents who were unable to fill out the questionnaire independently due to advanced age or physical conditions, an oral narration and recording approach was adopted. This ensured that the questionnaire was completed on their behalf with precision and meticulousness, based on a full understanding and respect for their intentions and expressions. Through this process, data required for the study was successfully collected.

### 2.3 Analytical framework

**Figure 1:**
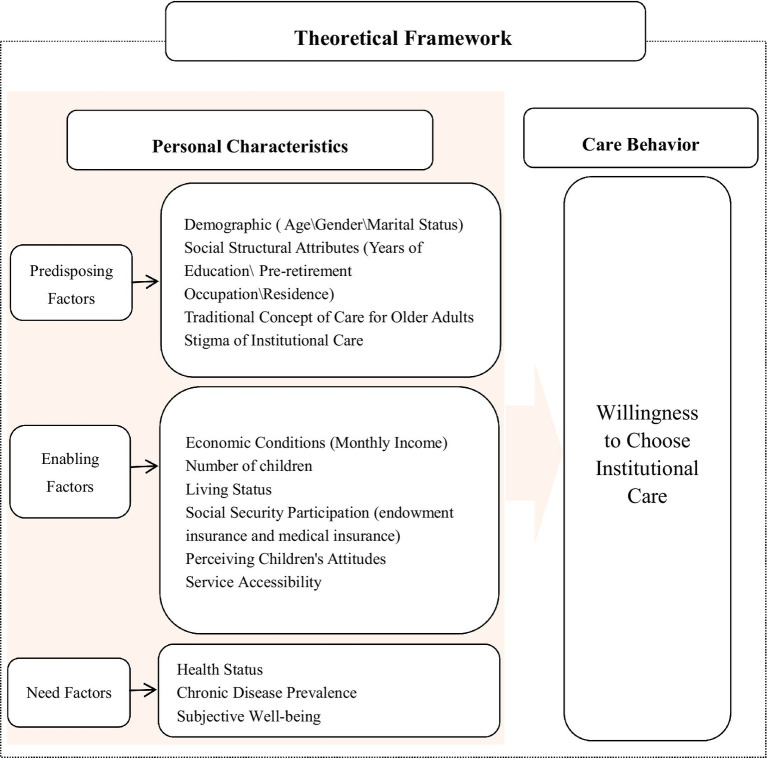
Analytical framework diagram of the Andersen model.

Based on the Andersen’s Model, this study designed the questionnaire by categorizing the influencing factors of the older adults’ care service selection into three types: predisposing factors, enabling factors, and need factors. This classification aims to systematize the research findings and facilitate the comparison of the influence among different categories of factors. Predisposing factors refer to the characteristics that make individuals with certain traits more inclined to choose a specific care service than others. These characteristics include: Demographic factors (age, gender, marital status); Social structural characteristics (years of education, pre-retirement occupation, place of residence); Traditional care concepts for older adults; Stigma of institutional care. Enabling factors denote the favorable conditions that help satisfy individuals’ needs for utilizing care services, including: Economic Conditions (Monthly Income); Number of children; Living Status; Social Security Participation (endowment insurance and medical insurance); Perceiving Children’s Attitudes; Service Accessibility. Need factors are the direct factors that trigger individuals’ needs for care services, including: Health Status; Chronic Disease Prevalence; Subjective Well-being (see Figure 1 for details).

#### 2.3.1 Dependent variables

In this study, the dependent variable is defined as the older adults’ willingness to receive institutional care, which is a binary variable. It is coded as “0” when the older adults are unwilling to receive institutional care, and “1” when they are willing to do so. This paper employs three types of indicators from the Andersen’s Model for data analysis, namely predisposing factors, enabling factors, and need factors. Prior to conducting the data analysis, the survey data were classified and assigned values, with the specific details presented in Table 1.

**Table 1:** Variable assignment details.

Variable name	Variable category	Meaning or assignment
Willingness to receive institutional care	Categorical variable	0 = Unwilling; 1 = Willing
Predisposing factors		
Gender	Categorical variable	0 = Female; 1 = Male
Age	Continuous variable	Self-reported age
Residence	Categorical variable	0 = Rural; 1 = Urban
Marital status	Categorical variable	0 = Unmarried, divorced, or widowed; 1 = Married
Years of education	Continuous variable	Self-reported
Pre-retirement occupation	Categorical variable	1 = Unemployed; 2 = Farmer; 3 = Enterprise employee; 4 = Staff of government agencies or public institutions; 5 = Individual service worker
Traditional concept of care for older adults: some individuals think that “having children is a means to secure support in old age.” What is your opinion on this perspective?	Categorical variable	0 = Oppose; 1 = Support
Stigma of Institutional Care: some individuals believe that placing parents in a nursing home is undutiful to them. What is your opinion on this perspective?	Categorical variable	0 = Oppose; 1 = Support
Enabling factors		
Monthly income	Continuous variable	Self-reported
Number of children	Continuous variable	Self-reported
Living status	Categorical variable	0 = Living alone; 1 = Not living alone
Endowment insurance	Categorical variable	0 = Not participating; 1 = Participating
Medical insurance	Categorical variable	0 = Not participating; 1 = Participating
Perceiving children’s attitudes: children’s willingness toward your institutional care?	Categorical variable	0 = Negative; 1 = Positive
Service accessibility: any care institutions for older adults near your residence?	Categorical variable	0 = No; 1 = Yes
Need factors		
Self-rated health status	Continuous variable	1 = Excellent: Capable of caring for others; 2 = Good: Generally do not require care from others; 3 = Fair: Need assistance with certain tasks; 4 = Poor: Fully dependent on others for care
Chronic disease status	Categorical variable	0 = No chronic diseases; 1 = Having one or more chronic diseases
Life satisfaction	Continuous variable	1 = Very satisfied; 2 = Relatively satisfied; 3 = Neutral; 4 = Not very satisfied; 5 = Very dissatisfied

#### 2.3.2 Independent variables

The influencing factors of the older adults’ willingness toward institutional care were categorized into three secondary indicators: Predisposing factors, enabling factors, and need factors, which were further refined into 18 tertiary indicators (see Table 1 for measurement details).

#### 2.3.3 Statistical analysis

To conduct an in-depth analysis of the basic conditions of the older adults and the distribution of their willingness toward institutional care, this study first employed descriptive statistical methods for preliminary exploration. Subsequently, a chi-square test was used to examine key influencing factors through univariate analysis. On this basis, to reveal the multiple factors affecting the older adults’ willingness to accept institutional care, a binary logistic regression model was adopted in this research, with the willingness toward institutional care as the dependent variable and the three core factors as independent variables.

In the process of model construction for this study, a baseline Model 1 was first established, which included predisposing factors as control variables. Subsequently, with the aim of conducting a more comprehensive investigation into the influencing factors, enabling factors and need factors were gradually incorporated to form Model 2 and Model 3, respectively. The expressions for these three models are presented as follows:

Model 1: Logit(Yi) = Predisposing Factors

Model 2: Logit(Yi) = Predisposing Factors + Enabling Factors

Model 3: Logit(Yi) = Predisposing Factors + Enabling Factors + Need Factors

To ensure the accuracy and reliability of data processing, this study utilized the SPSS 25.0 statistical software for handling all data. Meanwhile, the two-tailed test principle was adopted, with a significance level of *α* = 0.05.

## 3 Results

### 3.1 Model testing

Collinearity diagnosis and goodness-of-fit test were conducted for the variables. To avoid collinearity among variables, a collinearity test was performed. The results showed that the Variance Inflation Factor (VIF) values of all variables were below 2 (ranging from 1.103 to 2.579), which were far lower than the critical value of 5 (22). This indicates that there was no multicollinearity in the model, and the regression results were robust. SPSS 25.0 was used to conduct goodness-of-fit tests on the model. It was found that the fitting effect improved progressively from Model 1 to Model 3. For the final model, the chi-square value was 244.268 with a significance *p*-value of 0.000, indicating statistical significance. The Nagelkerke *R*^2^ was 0.593 (>0.2), which suggests that the model had high explanatory power (23). For the Hosmer–Lemeshow Test, the chi-square value was 5.964 and *p* = 0.651 (>0.05), indicating a good model fit (24). Model fit coefficients are presented in Table 2.

**Table 2:** Model fit coefficients.

Parameters		Model 1	Model 2	Model 3
Omnibus test		108.091***	223.564***	244.268***
Nagelkerke *R*^2^		0.306	0.556	0.593
AIC		492.679	391.206	376.502
BIC		545.047	471.771	469.152
Hosmer–Lemeshow test	*X* ^2^	12.872	8.049	5.964
	*p*	0.116	0.429	0.651

*** represent significance levels of 1%.

### 3.2 Basic attributes of the sample

#### 3.2.1 Predisposing factors attributes of older adults

Table 3 presents the attributes of predisposing factors for the sample in this study. Among these attributes, the gender ratio was relatively balanced, with a slightly larger proportion of females than males: males accounted for 49.64%, and females for 50.36%. The average age was 69.627 years. A total of 76.39% of the older adults had a spouse. The average number of years of education was 9.11 years. Regarding pre-retirement occupations, enterprise employees constituted the largest group, with 147 individuals (accounting for 35.42% of the total sample). 42.9% of the older adults hold the traditional care concept of “having children is a means to secure support in old age,” whereas 52.3% of the older adults hold the perception of “stigma toward care institutions.”

**Table 3:** Predisposing factors attributes.

Variable	Category	Value	Composition ratio (%)
Gender	Male	206	49.64
	Female	209	50.36
Age	Mean	69.627	
Residence	Urban	219	52.77
	Rural	196	47.23
Marital status	Married	317	76.39
	Unmarried/divorced/widowed	98	23.61
Years of education	Mean	9.111	
Pre-retirement occupation	Unemployed	72	17.35
	Farmer	83	20.00
	Enterprise employee	147	35.42
	Staff of government agencies or public institutions	43	10.36
	Individual service worker	63	15.18
	Others	7	1.69
Traditional concept of care for older adults	Support	178	42.9
	Oppose	237	57.1
Stigma of institutional care	Support	217	52.3
	Oppose	198	47.7

#### 3.2.2 Enabling factors attributes of older adults

Regarding enabling factors, the average monthly income of the surveyed older adults was 2,124.096 yuan. The average number of children per older adult was 1.665. Approximately 74.70% of the older adults lived with their spouses and/or children. Over 60% of the older adults had endowment insurance, and about 87.47% had medical insurance. In terms of the older adults’ perception of their children’s attitudes toward institutional care, 22.17% of the older adults perceived a “positive” attitude, while 77.83% perceived a “negative” one. Regarding the accessibility of institutional care resources, 47.95% of the older adults reported convenient access, whereas 52.05% reported inconvenient access (see Table 4 for the specific distribution).

**Table 4:** Enabling factors attributes.

Variable	Category	Value	Composition ratio (%)
Monthly income	Mean	2124.096	
Number of children	Mean	1.665	
Living status	Living alone	105	25.30
	Not living alone	310	74.70
Endowment insurance	Participating	260	62.65
	Not participating	155	37.35
Medical insurance	Participating	363	87.47
	Not participating	52	12.53
Perceiving children’s attitudes	Positive	92	22.17
	Negative	323	77.83
Service accessibility	Yes	199	47.95
	No	216	52.05

#### 3.2.3 Need factors attributes of older adults

The specific distribution of need factors is presented in Table 5. Approximately 68.67% of the older adults (285 individuals in total) had full self-care ability, while around 26.51% (110 individuals in total) had partial self-care ability, and roughly 4.82% (20 individuals in total) had no self-care ability at all. Over 70% of the older adults suffered from one or more chronic diseases, and about 55.66% expressed satisfaction with their current living conditions.

**Table 5:** Need factors attributes.

Variable	Category	Value	Composition ratio (%)
Self-rated health status	Excellent	95	22.89
	Good	190	45.78
	Fair	110	26.51
	Poor	20	4.82
Chronic disease status	No chronic diseases	116	27.952
	Having one or more chronic diseases	299	72.048
Life satisfaction	Very satisfied	94	22.65
	Relatively satisfied	137	33.01
	Neutral	96	23.13
	Not very satisfied	57	13.74
	Very dissatisfied	31	7.47

#### 3.2.4 Overall willingness of the older adults to receive institutional care

The survey results indicate that 44% of the older adults are willing to choose institutional care, while 56% are unwilling to do so.

### 3.3 Univariate analysis of institutional care willingness

#### 3.3.1 Impact of predisposing factors

Table 6 shows the impact of predisposing factors on the older adults’ willingness for institutional care. Independent samples t-test results indicated that there were significant differences in the willingness for institutional care among the older adults with different ages (*T* = −2.194, *p* = 0.029) and different years of education (*T* = 3.664, *p* = 0.000). Chi-square test results revealed a statistically significant difference in willingness across residential locations (*χ*^2^ = 8.165, *p* = 0.004), with urban-dwelling older adults showing stronger willingness. Additionally, significant differences were observed among older adults with different pre-retirement occupations (*χ*^2^ = 16.945, *p* = 0.005). The traditional care concept of “having children is a means to secure support in old age” also showed a significant relationship with institutional care willingness (*χ*^2^ = 48.866, *p* < 0.01), suggesting that traditional views on elder support influence preferences. Furthermore, a significant association was found between the stigma associated with nursing homes and willingness to use institutional care (*χ*^2^ = 72.940, *p* < 0.01). In contrast, factors such as marital status, gender, and age had no statistical significance (*p* > 0.05).

**Table 6:** The impact of predisposing factors on institutional care willingness.

Variable	Category	*N*	Institutional care willingness		*P*	*X*^2^ *T*
			Yes	No		
Gender	Male	206	95	111	0.411	0.677
	Female	209	88	121		
Age	Mean	415	68.86	70.45	0.029**	−2.194
Residence	Urban	219	111	108	0.004**	8.165
	Rural	196	72	124		
Marital status	Married	317	148	169	0.056	3.657
	Unmarried/divorced/widowed	98	35	63		
Years of education	Mean	415	8.26	9.902	0.000***	3.664
Pre-retirement occupation	Unemployed	72	20	52	0.005**	16.945
	Farmer	83	30	53		
	Enterprise employee	147	74	73		
	Staff of government agencies or public institutions	43	20	23		
	Individual service worker	63	34	29		
	Others	7	5	2		
Traditional concept of care for older adults	Oppose	237	158	79	0.000***	48.866
	Support	178	57	121		
Stigma of institutional care	Oppose	198	146	52	0.000***	72.940
	Support	217	69	148		

***, and ** represent significance levels of 1% and 5%, respectively.

#### 3.3.2 Impact of enabling factors

The results of the independent samples t-test showed that there were significant differences in the willingness for institutional care with respect to the variables of income (*T* = 2.989, *p* = 0.003) and number of children (*T* = −2.631, *p* = 0.009). Chi-square tests revealed that the association between living status and willingness to use institutional care was not statistically significant (*χ*^2^ = 0.019, *p* = 0.892). In contrast, a significant association was found between endowment insurance and willingness (*χ*^2^ = 73.803, *p* = 0.000), indicating that pension insurance has a statistically significant effect on preferences. Similarly, medical insurance status was significantly associated with willingness to use institutional care (*χ*^2^ = 17.111, *p* = 0.000). Perceived attitudes of adult children and accessibility of institutional care resources were also significantly associated with willingness (*p* = 0.000) (see Table 7).

**Table 7:** The impact of enabling factors on institutional care willingness.

Variable	Category	*N*	Institutional care willingness		*P*	*X*^2^/*T*
			Yes	No		
Economic income (*Z*-score standardization)	Mean	415	−0.151	0.14	0.003***	2.989
Number of children	Mean	415	1.54	1.8	0.009***	−2.631
Living status	Living alone	105	34	71	0.892	0.019
	Not living alone	143	68	75		
Endowment insurance	Participating	260	177	38	0.000***	73.803
	Not participating	155	83	117		
Medical insurance	Participating	363	202	161	0.000***	17.111
	Not participating	52	13	39		
Perceiving children’s attitudes	Negative	323	131	192	0.000***	73.857
	Positive	92	84	8		
Service accessibility	No	216	55	161	0.000***	125.213
	Yes	199	160	39		

*** represent significance levels of 1%.

#### 3.3.3 Impact of need factors

In the study of need factors, chi-square tests revealed that the older adults’ self-rated health status had a significant impact on their willingness to use institutional care (*p* = 0.000). Furthermore, the presence of chronic disease was also significantly associated with institutional care preference (*p* = 0.005), revealing notable differences in willingness based on chronic disease status. In addition, life satisfaction was found to be significantly associated with willingness to adopt institutional care (*p* = 0.000), suggesting that higher or lower levels of life satisfaction considerably affect older adults’ preferences for institutional care (see Table 8 for details).

**Table 8:** The impact of enabling factors on institutional care willingness.

Variable	Category	*N*	Institutional care willingness		*P*	*X* ^2^
			Yes	No		
Self-rated health status	Excellent	95	48	47	0.000***	55.331
	Good	190	111	79		
	Fair	110	24	86		
	Poor	20	0	20		
Chronic disease status	No chronic diseases	299	157	142	0.005***	7.979
	Having one or more chronic diseases	116	73	43		
Life satisfaction	Very satisfied	94	35	59	0.000***	44.603
	Relatively satisfied	137	83	54		
	Neutral	96	46	50		
	Not very satisfied	57	19	38		
	Very dissatisfied	31	0	31		

*** represent significance levels of 1%.

### 3.4 Logistic regression analysis of institutional care willingness

After in-depth analysis of the data, this study constructed three regression models to explore the correlation between different factors and the willingness for institutional care (see Table 9 for measurement details).

**Table 9:** Logistic regression analysis of institutional care willingness.

Independent variable	Model 1		Model 2		Model 3	
	Regression coefficient (standard error)	OR (95%CI)	Regression coefficient (standard error)	OR (95%CI)	Regression coefficient (standard error)	OR (95%CI)
Constant term	2.233 (1.238)	9.329	−0.186 (1.519)	0.830	−2.270 (1.803)	0.103
Gender						
Female	Referent	Referent	Referent	Referent	Referent	Referent
Male	−0.070 (0.234)	0.932 (0.589, 1.475)	0.069 (0.285)	1.072 (0.612, 1.875)	−0.006 (0.300)	0.994 (0.552, 1.789)
Age	−0.022 (0.016)	0.978 (0.948, 1.009)	−0.013 (0.018)	0.987 (0.952, 1.023)	−0.010 (0.019)	0.990 (0.953, 1.029)
Residence						
Rural	Referent	Referent	Referent	Referent	Referent	Referent
Urban	0.189 (0.233)	1.208 (0.765, 1.906)	−0.184 (0.285)	0.832 (0.476, 1.454)	−0.366 (0.302)	0.694 (0.384, 1.253)
Marital status						
Married	Referent	Referent	Referent	Referent	Referent	Referent
Unmarried/divorced/ widowed	0.147 (0.284)	1.159 (0.664, 2.020)	−0.438 (0.379)	0.645 (0.307, 1.356)	−0.514 (0.390)	0.598 (0.279, 1.284)
Years of education	0.067*(0.026)	1.069 (1.015, 1.126)	0.005 (0.032)	1.005 (0.944, 1.070)	−0.004 (0.033)	0.996 (0.934, 1.063)
Occupation before retirement						
Unemployed	Referent	Referent	Referent	Referent	Referent	Referent
Farmer	−0.232 (0.389)	0.793 (0.370, 1.701)	0.147 (0.498)	1.159 (0.437, 3.073)	0.099 (0.503)	1.104 (0.412, 2.961)
Enterprise employee	−0.037 (0.349)	0.963 (0.486, 1.910)	−0.034 (0.450)	0.967 (0.400, 2.335)	0.061 (0.469)	1.063 (0.424, 2.664)
Staff of government agencies or public institutions	0.197 (0.460)	1.218 (0.494, 3.001)	0.105 (0.579)	1.111 (0.357, 3.456)	0.116 (0.604)	1.122 (0.343, 3.669)
Individual service worker	0.377 (0.412)	1.458 (0.650, 3.272)	0.422 (0.516)	1.524 (0.554, 4.194)	0.408 (0.538)	1.503 (0.524, 4.313)
Others	−0.180 (0.860)	0.835 (0.155, 4.504)	−1.312 (1.091)	0.269 (0.032, 2.287)	−1.527 (1.149)	0.217 (0.023, 2.065)
Traditional concept of care for older adults						
Oppose	Referent	Referent	Referent	Referent	Referent	Referent
Support	−0.981***(0.244)	0.375 (0.232, 0.605)	−0.372 (0.309)	0.690 (0.377, 1.263)	−0.435 (0.327)	0.647 (0.341, 1.228)
Stigma of institutional care						
Oppose	Referent	Referent	Referent	Referent	Referent	Referent
Support	−1.448***(0.238)	0.235 (0.147, 0.375)	−1.072***(0.288)	0.342 (0.194, 0.602)	−1.102***(0.301)	0.332 (0.184, 0.599)
Monthly income			0.145 (0.154)	1.156 (0.854, 1.565)	0.118 (0.162)	1.125 (0.819, 1.547)
Number of children			−0.199 (0.153)	0.820 (0.607, 1.107)	−0.164 (0.162)	0.849 (0.618, 1.165)
Living arrangement						
Living alone	Referent	Referent	Referent	Referent	Referent	Referent
Not living alone			−0.717*(0.400)	0.488 (0.223, 1.070)	−0.555 (0.413)	0.574 (0.256, 1.289)
Endowment insurance						
Not participating	Referent	Referent	Referent	Referent	Referent	Referent
Participating			1.015***(0.317)	2.759 (1.482, 5.136)	0.860**(0.334)	2.363 (1.228, 4.550)
Medical insurance						
Not participating	Referent	Referent	Referent	Referent	Referent	Referent
Participating			0.793 (0.553)	2.210 (0.748, 6.528)	1.241**(0.545)	3.458 (1.188, 10.068)
Perceiving children’s attitudes						
Negative	Referent	Referent	Referent	Referent	Referent	Referent
Positive			2.128***(0.461)	8.399 (3.405, 20.720)	1.935***(0.465)	6.921 (2.783, 17.215)
Service accessibility						
No	Referent	Referent	Referent	Referent	Referent	Referent
Yes			1.501***(0.289)	4.487 (2.548, 7.900)	1.286***(0.304)	3.619 (1.995, 6.563)
Self-rated health status					0.568**(0.224)	1.766 (1.139, 2.736)
Chronic disease status						
No	Referent	Referent	Referent	Referent	Referent	Referent
Yes					−0.643*(0.352)	0.526 (0.264, 1.048)
Life satisfaction					0.150 (0.155)	1.162 (0.858, 1.574)

***, **, and ** represent significance levels of 1% and 5%, and 10%, respectively.

Model 1 significantly revealed the direct impact of educational level, traditional concept of care for older adults, stigma of institutional care (among predisposing factors) on the willingness for institutional care. Specifically, educational level had a significant positive effect on the willingness for institutional care. For each additional unit increase in educational attainment, the older adults’ willingness to utilize institutional care increased by 6.9% (*β* = 0.067, *p* < 0.1; OR = 1.069). This may be attributed to the fact that higher education levels facilitate better access to and understanding of information related to institutional care. Further analysis revealed that traditional care concept and the stigma associated with institutional care exerted significant negative effects. Older adults holding traditional care concepts to choose institutional care decreased by 62.5% (*β* = −0.981, *p* < 0.01; OR = 0.375), while the willingness of those who perceived “sending older adults to nursing homes as being unfilial” to choose institutional care decreased by 76.5% (*β* = −1.448, *p* < 0.01; OR = 0.235). In contrast, factors such as gender, age, place of residence, marital status, and pre-retirement occupation did not exhibit statistically significant effects on willingness to use institutional care in the model.

In Model 2, the variables of enabling factors were introduced. Among these enabling factors, participation in endowment insurance, perceived children’s attitudes toward institutional care, and accessibility of care resources were confirmed to be significant factors affecting the willingness for institutional care. However, years of education and traditional care concepts in Model 1 no longer had a significant impact on this willingness. Specifically, compared with the group not participating in endowment insurance, the willingness for institutional care among older adults with endowment insurance was 2.759 times higher (*β* = 1.015, *p* < 0.01; OR = 2.759). In addition, living status exerted a significant negative impact on willingness: non-living-alone older adults were 51.20% less willing than those living alone (*β* = −0.717, *p* < 0.1; OR = 0.488). Furthermore, older adults’ perceived positive attitudes of their children toward institutional care (*β* = 2.128, *p* < 0.01; OR = 8.399) and accessibility of institutional care resources (*β* = 1.501, *p* < 0.01; OR = 4.487) had a positive impact on their willingness for institutional care. Older adults with children’s support were 8.399 times more willing, and those with nearby care institutions were 4.487 times more willing. However, variables including monthly income, number of children, and participation in medical insurance showed no significant effects in Model 2.

In the construction of Model 3, need factors were introduced as a consideration. When controlling for predisposing factors and enabling factors, the following variables remained significantly and stably associated with older adults’ willingness for institutional care: stigma toward institutional care (*β* = −1.102, *p* < 0.01; OR = 0.332), having old-age insurance (*β* = 1.527, *p* < 0.01; OR = 4.602), perceived positive attitudes of children (*β* = 1.935, *p* < 0.01; OR = 6.921), accessibility of institutional care services (*β* = 1.286, *p* < 0.01; OR = 3.619). However, living status (whether the older adults live alone or not)—a variable categorized under enabling factors in Model 2—no longer exerted a significant impact on the willingness for institutional care in Model 3. With the inclusion of need factors, further analysis revealed that the older adults’ self-rated health status had a significant positive impact on their willingness for institutional care. Specifically, for each unit increase in self-rated health, the willingness increased by 1.766 times (*β* = 0.568, *p* < 0.05; OR = 1.766). Meanwhile, having chronic diseases exerted a significant negative impact on the willingness (*β* = −0.643, *p* < 0.1; OR = 0.526), meaning that the willingness of older adults with chronic diseases to choose institutional care was 47.4% lower than that of those without chronic diseases. Additionally, the ratio of the probability of older adults with medical insurance being willing to move into institutional care to that of those without medical insurance was 3.458 (*β* = 1.241, *p* < 0.05; OR = 3.458), indicating that having medical insurance enhanced the older adults’ willingness for institutional care. In Model 3, life satisfaction showed no significant impact on the willingness for institutional care.

## 4 Discussion

### 4.1 Key findings of this study

A cross-sectional study design was adopted to examine the factors influencing Chinese older adults’ willingness to use institutional care. The study found that 44% of the older adults were willing to accept institutional care, which was higher than the 31.95% result from the 2014 “China Longitudinal Aging Social Survey (CLASS)” data (6). This indicates that with the passage of time, the care concept among Chinese people is gradually changing. Currently, home-based care services in Jiangning District of Nanjing have taken a leading role in innovation and implementation. For instance, relying on the district-level “Internet + Nursing Home” initiative, Jiangning has further developed the “Xiaojiang Home Care” brand for home-based care for older adults. By leveraging information technology and the Internet of Things (IoT), it has built an information-based, large-scale, and brand-oriented home care service system. This initiative has been recognized as a national exemplary case in the pilot reform of care services. Meanwhile, Jiangning District has piloted innovative models such as the “Rice Field Care Commune” in Hushu Sub-district and “Homestay Mutual Assistance Care” in Guli Sub-district, forming a “one village, one brand” service pattern. Throughout this process, the government has fully played a leading role: on the basis of ensuring that care service funds are included in the fiscal budget, it has introduced various social organizations, fostering a multi-stakeholder participation framework for older adults’ care. Nevertheless, survey results indicate a relatively high willingness among the older adults in the district to choose institutional care—even higher than the findings of previous studies. This further demonstrates that the older adults’ care willingness is multi-dimensional and dynamically changing: willingness for home-based care represents their “current preference,” while willingness for institutional care may reflect a “future-oriented.” To a certain extent, this also reflects the proactive planning awareness of the older adults in Jiangning District regarding potential care risks in the future.

The main findings are as follows: (1) among the predisposing factors, the stigma associated with institutional care significantly reduces the willingness of older adults to engage in institutional care. (2) The key determinants of older adults’ willingness to choose institutional care are enabling factors and need factors. Specifically, older adults who have endowment insurance, medical insurance, perceive their children as holding a more positive attitude toward institutional care, have easier access to institutional care resources, and report better self-rated health demonstrate a significantly stronger willingness to select institutional care. (3) The presence of chronic diseases significantly decreases the willingness of older adults to opt for institutional care.

Among the predisposing factors, when only the influence of predisposing factors is considered, educational level emerges as a significant determinant. This finding aligns with the results of a previous study (25). This study concludes that older adults with higher educational attainment exhibit a stronger inclination toward accepting institutional care. Generally, older adults with higher education are more receptive to new things and new care models. They may be more open-minded and willing to try institutional care—a new type of care model. Such older adults often have relatively high emotional needs, yet they also hope to maintain a certain degree of independence and freedom. Social activities and companionship with peers in institutions help reduce loneliness and improve quality of life. Institutional care can provide an environment where they can not only enjoy friendship and family affection but also maintain an independent lifestyle. However, regarding differences between urban and rural residential areas, this study did not show an impact on the willingness for institutional care, which differs from previous studies. (25). This may be attributed to the narrowed urban–rural gap in Nanjing, a developed city in eastern China.

In Model 1, the predisposing factors further incorporate older adults’ personal care attitudes and perceptions toward institutional care. Older adults who hold traditional care concept demonstrate lower willingness to choose institutional care, a finding consistent with previous research (26). This implies that in daily life, these older adults tend to seek more psychological care, financial support, and physical assistance from their children; consequently, they reduce their demand for institutional care and instead opt for home-based care (7). However, the influence of traditional attitudes becomes insignificant when combined with the effects of other factors. More importantly, the stigma associated with institutional care maintains a consistent and robust negative impact across subsequent models, which aligns with prior studies (10, 27). “Filial piety” is a core concept in Confucianism, and the stigma of institutional care originates from China’s filial piety culture—under this cultural framework, children are expected to directly assume the responsibility of supporting their parents, including showing respect and obedience to them. When older adults consider choosing institutional care, they may face significant psychological pressure derived from social stigma: they perceive admission to a care institution as a sign of being abandoned by their children. Thus, this stigma associated with institutional care reduces older adults’ willingness to engage in institutional care.

Among enabling factors, endowment insurance emerges as a significant factor. This study finds that older adults with endowment insurance exhibit a stronger willingness to choose institutional care, which contradicts the findings of a study conducted in Gansu Province, China. Wang et al. (28) argued that endowment insurance provides older adults with crucial financial security and exerts a certain “substitution effect”: the sense of security and satisfaction derived from this insurance reduces their demand for institutional care services. According to the China Statistical Yearbook 2024 (29), Jiangsu Province had 37.52 million participants in the basic urban employee endowment insurance program in 2023, comprising 26.46 million active employees and 11.06 million retirees. In contrast, Gansu Province reported 5.325 million participants in the same program, including 3.524 million active employees and 1.801 million retirees. For the basic urban–rural resident endowment insurance scheme, Jiangsu had 23.519 million participants, with 11.445 million actually receiving benefits. Meanwhile, Gansu recorded 13.79 million participants in this program, of whom 3.514 million were benefit recipients. In terms of the number of benefit recipients, Jiangsu Province had significantly more older adult benefit claimants. Regarding the proportion of recipients: in Jiangsu, urban employee pension recipients accounted for 49.1% of the total beneficiaries, while urban–rural residential pension recipients made up 50.9%; in Gansu, the corresponding proportions were 33.9% (urban employee) and 66.1% (urban–rural residential). Compared with residential pensions, urban employee pensions provide higher purchasing power. Residential pensions primarily serve as a basic guarantee and are barely sufficient to cover the costs of institutional care services. Therefore, regions with a larger number and higher proportion of urban employee pension recipients tend to have older adult populations with stronger willingness and capacity to choose institutional care. When older adults have more stable financial resources, they can afford the expenses of institutional care and demonstrate a higher willingness to pay for such services. Institutional care typically offers comprehensive services, including accommodation, meals, medical care, and nursing care—costs that are relatively manageable for older adults with endowment insurance in eastern China (e.g., Jiangsu Province).

Whether older adults live alone emerged as a significant factor in Model 2, though its significance diminished when the joint effects of subsequent factors were considered. This influencing factor has also been identified in previous studies (30, 31). Compared with older adults who do not live alone, those living alone have less companionship and care from family members. Institutional care, however, plays a substitutive role by providing professional care and companionship services; consequently, older adults living alone exhibit a higher willingness to choose institutional care.

Older adults’ perceived positive attitudes of their children toward institutional care and their easier access to institutional care resources emerged as significant factors. For older adults, their perception of their children’s positive or negative attitudes toward institutional care directly influences their own choice of institutional care—a finding consistent with the research results of Broyles et al. (32). If adult children lack the capacity to provide long-term care for their parents and hold negative attitudes toward institutional care, this will naturally reduce older adults’ willingness to choose institutional care. Conversely, when older adults perceive the importance, accessibility, and functionality of institutional care in their vicinity, their willingness to opt for such care increases accordingly (33). Meanwhile, when care institutions are relatively close to their homes, it also facilitates adult children’s ability to visit and care for their parents at any time.

Among need factors, self-rated health status and chronic diseases status were significant factors. Different from previous studies which found that self-rated health status had no impact on the willingness for institutional care (31, 34), this study concluded that older adults who rated their own health as better were more willing to move into care institutions. Practical surveys of such institutions also support this finding: the proportion of self-care capable older adults in current care institutions is considerable. Taking Yincheng Kangyang Care Service for Older Adults Co., Ltd., a local brand care enterprise in Nanjing, as an example, this enterprise operates 30 care institutions in Nanjing. Among its comprehensive care institutions with over 210 beds, self-care capable older adults account for approximately 25%. In some other public care institutions, the proportion of self-care capable older adults is even higher. Compared with older adults in poor health, who have no choice but to accept a specific care model, those in good health have greater autonomy in planning their later-life care. Consequently, institutional care has increasingly become a preferred option for a growing number of healthy older adults. From the perspective of institutional care in Jiangning District: the district has introduced professional care institutions to provide custody services, with the District Social Welfare Center and 8 sub-district-level older adult homes all operated by branded care service organizations. Additionally, 23 community health service centers in the district have been registered as care institutions—these measures have effectively improved the professionalization level of care institutions. The professional services of these institutions further attract older adults in good health to choose institutional care. Furthermore, among the 33 care institutions in the district, many focus on preventive health preservation services, functioning as living communities that emphasize social and health-related activities for older adults, rather than being simple traditional nursing facilities. For instance, the care institutions under Yuehua Anyang in Jiangning District regularly organize various cultural and recreational activities, such as calligraphy and painting reading sessions, film screenings, and handicraft workshops, to enrich the spiritual life of older adults. Meanwhile, these institutions have set up psychological counseling rooms to provide psychological support and comfort for older adults, helping them maintain a positive and optimistic mindset. A number of care institutions have also established branch campuses of universities for older adults; for example, Jiangning Muchunyuan Nursing Home hosts a branch campus of Jiangning District University for Older Adults. Older individuals in good health may have a stronger expectation to pursue a higher quality of life and more diverse social activities in their later years. Institutional care typically offers a wide range of recreational, cultural, and social activities, which help older adults maintain vitality and achieve spiritual fulfillment. Older adults who perceive themselves as healthy may thus be more willing to try this new lifestyle. Institutional care provides an alternative to traditional home-based care, giving them the opportunity to experience a different living environment and social circle.

Older adults without chronic diseases exhibit a stronger inclination toward choosing institutional care, a finding consistent with other studies conducted in China (35), but contradicts the research by Mercedes Rodríguez in Spain (36). On the one hand, in China, older adults with chronic diseases face a heavier financial burden. On the other hand, although China is currently vigorously promoting the new “integrated medical and care” model—an approach that organically combines medical services with care services, providing professional medical and rehabilitation services in addition to traditional care services to integrate traditional care with modern medical services—this model is still in the exploratory stage (37). Older adults in China express distrust toward the limited medical services provided by care institutions. Consequently, those with chronic diseases tend to allocate their expenses to specialized chronic disease hospitals or other medical facilities to restore health, thereby exercising caution when considering institutional care as an option. In contrast, under Spain’s System of Autonomy and Care for Dependents (SAAD), all Spanish nationals and residents who have lived in the country for at least 5 years are entitled to receive either a subsidy or one of five types of long-term care (LTC) services—including preventive services, telemedicine, home care services, 24-h care, and institutional care—following an assessment of their dependency level. Older adults with chronic diseases in Spain require a long-term and stable living environment, and institutional care, as an accessible welfare option, can provide them with necessary services to support their future health and quality of life (36, 38).

More interestingly, after accounting for need factors—i.e., controlling for variables such as self-rated health status and chronic diseases, the factor of medical insurance (which was included in Model 2) became significant in Model 3 and exerted a positive impact on the willingness for institutional care. In the United States, medical insurance includes a nationwide health insurance program, while Medicaid provides a publicly funded safety net program that covers health care and long-term care (LTC) for low-income populations (39). China’s medical insurance primarily focuses on inpatient reimbursement. Although it does not cover the costs of staying in care institutions, having medical insurance means that older adults have relatively more disposable income and thus greater financial capacity to pay for the expenses of institutional care.

### 4.2 Limitations of this study

This study has several limitations. First, the probability-proportional sampling principle was not fully adopted. Although a multi-stage sampling method was employed, the high concentration and mobility of residents in urban communities made it difficult to obtain a completely accurate residential list as a sampling frame. Additionally, access control systems in many residential buildings and the specific daily schedules of older adults prevented the strict implementation of probability-proportional random sampling; thus, convenience sampling was used as a supplementary strategy. Consequently, the study findings are more suitable for explaining the intrinsic mechanism of relationships between influencing factors, rather than accurately estimating the overall proportion of older adults’ willingness to choose institutional care in Jiangning District or even Nanjing. Second, a cross-sectional survey design was adopted. The results can only reveal the correlation between variables, but not establish a rigorous causal relationship—given that all variables are in a dynamic state of development and change over time. Further research based on longitudinal data is therefore needed to confirm the causal relationships. Third, although the Andersen’s Model was applied with the inclusion of as many variables as possible, and culturally localized and innovative variables were incorporated, the factors influencing older adults’ personal decision-making are highly complex. Unmeasured confounding variables may still exist, such as older adults’ personality traits, digital literacy, and unobserved family dynamics. Fourth, biases may arise from subjective measurements. Variables such as traditional care attitudes, institutional care stigma, and perceived children’s attitudes were all measured based on self-reports, which may lead to measurement errors. Although optimizations were made during the questionnaire design phase to address subjective questions—for example, avoiding direct inquiries about sensitive issues and instead asking respondents whether they agreed with common viewpoints—such biases cannot be completely eliminated.

These limitations also point to directions for future research on this topic. For instance, future studies could: (1) expand the sample coverage to enhance representativeness; (2) adopt a longitudinal research design to further validate the study conclusions; and (3) incorporate in-depth interviews to collect data on deeper and more complex influencing factors.

## 5 Conclusion

Based on the Andersen’s Model, this study analyzed the willingness for institutional care among older adults in Jiangning District of Nanjing from three dimensions: predisposing factors, enabling factors, and need factors. An in-depth analysis was conducted on data collected through questionnaires. The results showed that the willingness of older adults in Jiangning District of Nanjing to accept institutional care was higher than that reported in existing studies, which may be largely attributed to the relatively sound regional care service system in the surveyed area.

The findings indicate that older adults who exhibit a stronger willingness to utilize institutional care are characterized by the following factors: openness to institutional care, possession of pension and health insurance, perception of more supportive attitudes from their adult children toward institutionalization, greater accessibility to institutional care resources, self-rated good health, and absence of chronic diseases.

This research offers an actionable guide for improving care system for older adults in Jiangning District. Currently, the local government has focused on addressing issues in the care service, formulating 13 supporting policies—including the Jiangning District Basic Care Service Guidance Catalog for Older Adults, standardized distribution of respect subsidies for older adults, standardized subsidy disbursement, home-based care beds for older adults, age-friendly home renovations, and the care “time bank” system—to promote the high-quality development of care services for older adults across the district. However, the existing policies tend to prioritize support for vulnerable groups such as those with severe or partial disabilities, while relatively less attention is given to healthy and active older adults. There is a need for the government to shift from a blanket “universal safety-net” approach toward more targeted guidance that responds to the diverse needs of different older adult populations. For instance, efforts could include promoting institutional care as a positive lifestyle choice to seniors with stable pensions, health insurance, and good health—as well as to their families—so as to reduce mistrust and negative perceptions of residential care facilities. When planning care facilities, greater attention should be paid to ensuring convenient access for older residents. Furthermore, existing policies should be optimized through measures such as issuing care vouchers and expanding the coverage of long-term care insurance, so as to stimulate demand and encourage broader participation in formal care services among diverse groups of older adults.

As the aging phenomenon becomes increasingly severe, the role of institutional care services in care service system is growing increasingly important, indicating extremely broad room for growth in the future. However, this finding does not imply that the development of the care system in Jiangning should prioritize institutional care at the expense of home-based models, nor should it encourage a blanket expansion of nursing facilities. Rather, the observed “high willingness toward institutional care” should be interpreted as a desire for high-quality, professional, and highly accessible care services for older adults in general. The ultimate goal remains to ensure that every older person can age with dignity and quality of life in the setting of their choice. This can be achieved by upgrading home- and community-based care, developing embedded neighborhood facilities, and building an integrated care network that offers flexible, continuous support. Rather than pitting “home-based” against “institutional” care, the emphasis should be on creating a diversified, responsive, and person-centered ecosystem of services.

## Data Availability

The raw data supporting the conclusions of this article will be made available by the authors, without undue reservation.
